# Distinct growth patterns in seedling and tillering wheat plants suggests a developmentally restricted role of HYD2 in salt-stress response

**DOI:** 10.1007/s00299-024-03206-x

**Published:** 2024-04-17

**Authors:** Cody Bekkering, Shu Yu, Chih Chi Kuo, Li Tian

**Affiliations:** Department of Plant Sciences, University of California, Davis, CA 95616 USA

**Keywords:** Wheat, Salt-stress response, Salinity, Carotenoid, Carotenoid β-hydroxylase (HYD), β-Carotene, Hydroxylation, Xanthophyll

## Abstract

**Key message:**

Mutants lacking functional HYD2 homoeologs showed improved seedling growth, but comparable or increased susceptibility to salt stress in tillering plants, suggesting a developmentally restricted role of HYD2 in salt response.

**Abstract:**

Salinity stress threatens global food security by reducing the yield of staple crops such as wheat (*Triticum* ssp.). Understanding how wheat responds to salinity stress is crucial for developing climate resilient varieties. In this study, we examined the interplay between carotenoid metabolism and the response to salt (NaCl) stress, a specific form of salinity stress, in tetraploid wheat plants with mutations in *carotenoid β-hydroxylase 1* (*HYD1)* and *HYD2*. Our investigation encompassed both the vulnerable seedling stage and the more developed tillering stage of wheat plant growth. Mutant combinations lacking functional HYD2 homoeologs, including *hyd-A2 hyd-B2, hyd-A1 hyd-A2 hyd-B2, hyd-B1 hyd-A2 hyd-B2*, and *hyd-A1 hyd-B1 hyd-A2 hyd-B2*, had longer first true leaves and slightly enhanced root growth during germination under salt stress compared to the segregate wild-type (control) plants. Interestingly, these mutant seedlings also showed decreased levels of neoxanthin and violaxanthin (xanthophylls derived from β-carotene) and an increase in β-carotene in roots. However, tillering *hyd* mutant and segregate wild-type plants generally did not differ in their height, tiller count, and biomass production under acute or prolonged salt stress, except for decreases in these parameters observed in the *hyd-A1 hyd-B1 hyd-A2 hyd-B2* mutant that indicate its heightened susceptibility to salt stress. Taken together, these findings suggest a significant, yet developmentally restricted role of HYD2 homoeologs in salt-stress response in tetraploid wheat. They also show that *hyd-A2 hyd-B2* mutant plants, previously demonstrated for possessing enriched nutritional (β-carotene) content, maintain an unimpaired ability to withstand salt stress.

**Supplementary Information:**

The online version contains supplementary material available at 10.1007/s00299-024-03206-x.

## Introduction

Wheat (*Triticum* spp.) plays an important role in the global food system, providing ~20% of the world’s caloric intake. In addition, the demand for wheat is projected to increase by 40% by 2050 due to global population growth (Shiferaw et al. 2013). However, the ongoing climate change poses a significant threat to wheat production and global food security by exacerbating environmental stressors such as salinity (van Zelm et al. 2020; Zörb et al. 2019). Salinity interferes with the growth and development of wheat plants, similar to other crops, by reducing water potential, disrupting ion balance, impairing protein function, and increasing the production of reactive oxygen species (ROS) that disrupt cellular functions (EL Sabagh et al. 2021). In agricultural soils, sodium chloride (NaCl) concentrations as low as 40 mM are considered saline, with concentrations around 100 mM reportedly causing a 50% reduction in dry mass in tetraploid wheat (Colmer et al. 2005). Identifying salinity-tolerant germplasm and developing diverse strategies to enhance metabolic processes in wheat plants under saline conditions holds promise in mitigating this globally relevant threat to food security.

Plants have developed various mechanisms to thrive in high-salinity environments, including salt exclusion and secretion, osmotic adjustments, salt-ion sequestration and compartmentalization, as well as the production of antioxidants to combat oxidative stress induced by salinity (van Zelm et al. 2020). These processes are tightly regulated by phytohormones, with one particularly important phytohormone being abscisic acid (ABA). ABA, synthesized from carotenoids, plays a central role in enhancing plant salinity tolerance by modulating many of the aforementioned processes (van Zelm et al. 2020; Yu et al. 2020). Interestingly, another class of carotenoid-derived phytohormone, strigolactones (SLs), is also suggested to affect salinity tolerance in plants (Yu et al. 2020). For example, *Arabidopsis thaliana* mutant plants defective in SL biosynthesis or signaling have exhibited reduced tolerance to salt (NaCl) stress, a form of salinity stress (Ha et al. 2014), whereas *A. thaliana* plants that overexpress *more axillary growth 2* (*MAX2*), a central component of SL signaling in plant cells, have displayed elevated salt tolerance (Wang et al. 2019).

Carotenoids, the biosynthetic precursors for ABA and SLs, are well known for their antioxidant capacities and have also been implicated in influencing the response of plants to salinity (Fig. S1) (Gómez-Sagasti et al. 2023). Several studies have investigated the connection between carotenoid accumulation in sweet potato (*Ipomoea batatas*) and its response to salt stress by manipulating carotenoid levels in plant tissues. Through altering the expression levels of *β-hydroxylase, lycopene β-cyclase*, *lycopene ε-cyclase,* and a mutant form of *Orange* (*Or*) using RNA interference (RNAi) or overexpression, increased accumulation of total carotenoids and enhanced salt tolerance were observed in sweet potato calli, cultured cells, and mature transgenic plants (Kang et al. 2018, 2017; Ke et al. 2019; Kim et al. 2019, 2012, 2014, 2013). Overexpression of *carotenoid β-hydroxylase* in *A. thaliana* and *lycopene β-cyclase* in tomato (*Solanum lycopersicum*) also led to increased total carotenoids and improved tolerance to salt stress (Davison et al. 2002; Mi et al. 2022). However, there is a lack of studies assessing the relationship between changes in carotenoid accumulation in wheat and its response to salinity stress.

In our efforts to dissect the role of carotenoid metabolic genes in wheat plant growth and development, we previously generated and characterized a series of tetraploid wheat mutant combinations, collectively termed *hyd* mutants, which contain lesions in *carotenoid β-hydroxylase 1* and *2* (designated *HYD1* and *HYD2* in wheat) homoeologs (Bekkering et al. 2023). HYDs carry out the hydroxylation of β-rings in carotenoid substrates, including the conversion of β-carotene to β-xanthophylls (Fig. S1). Our study revealed that HYD1 homoeologs significantly influence carotenoid profiles and photosynthesis in shoots of tetraploid wheat plants, while HYD2 homoeologs play a greater role in grain carotenoid accumulation, particularly in the hydroxylation of β-carotene (Bekkering et al. 2023). Despite changes to carotenoid profiles in multiple tissues, when not subjected to stress treatments, these mutant lines displayed growth and photosynthetic phenotypes similar to the segregate wild type, which is a sister line in the segregating population that is wild type for the *HYD1* and *HYD2* homoeologs (Bekkering et al. 2023; Yu et al. 2022b). An exception was observed in *hyd-A1 hyd-B1 hyd-A2 hyd-B2*, the quadruple mutant lacking all HYD activities, which exhibited reduced photoprotective responses and a slight developmental delay (Bekkering et al. 2023; Yu et al. 2022b).

It is important to note that while previous studies have primarily focused on examining salt tolerance in mature plants with modified carotenoid profiles, salt stress affects plant growth at all stages of development, including during seed germination and seedling growth when the plant is particularly vulnerable to environmental stress (El Sabagh et al. 2021; Maas and Poss 1989). There is a significant knowledge gap concerning the impact of salt on seedling growth in wheat and the potential role of carotenoids in modulating the response of wheat to salt stress during this critical stage of plant development. To address this knowledge gap, our current study leveraged the combinatorial *hyd* mutants with varying carotenoid concentrations in different tissues and conducted a suite of phenotypic analyses in seedlings under salt-stress conditions. Additionally, carotenoid concentrations in roots and first true leaves of these wheat seedlings were quantified. Furthermore, in the more advanced tillering stage of plant development, phenotypic traits including biomass, tiller count, and shoot water fraction were determined in these mutants when they were exposed to salt stress in both soil- and hydroponic-based growth systems.

## Materials and methods

### Salt-stress treatments and phenotyping of wheat seedlings

Seeds of *hyd* mutants and the segregate wild type were surface-sterilized using a 1.0% (v/v) commercial bleach solution (8.25% sodium hypochlorite prior to the 1:100 dilution) and rinsed at least 5 times with distilled water. To examine the susceptibility of wheat seeds to salt stress during imbibition, the lengths of roots as well as first true leaves of Kronos (the wild-type parent of *hyd* mutants) seedlings were measured for seeds germinated under varying salt levels (0, 100 or 200 mM NaCl). For each salt level, one half of the seeds were initially imbibed in a dish of deionized (DI) water and then transferred to germination paper with the corresponding salt level, while the other half were imbibed on the same germination paper (with the same salt concentration) on which they would eventually germinate. In a separate analysis, seeds that were imbibed in a petri dish containing 200 mM NaCl and then transferred to germination paper containing 0 mM NaCl (i.e., DI water) were compared to seeds that imbibed and germinated in DI water.

For salt-stress treatments, sterilized seeds were placed in transparent, standard-weight sheet protectors (Office Depot, Boca Raton, FL) on a sheet of germination paper (Blue Blotter 76#, Anchor Paper Co., St. Paul, MN) soaked with 40 mL of sterile DI water or salt solution. For the pilot experiment with the segregate wild type, *hyd-A1 hyd-B1*, and *hyd-A2 hyd-B2* genotypes, the following salt solutions were used: 0, 50, 100, 150, and 200 mM NaCl. For phenotypic analyses comparing the salt-stress response of combinatorial *hyd* mutants and the segregate wild-type line, 100 mM NaCl was applied. The germination paper was positioned at the bottom of the sheet protector (collectively referred to as a germination sleeve). Seeds were placed at the midpoint of the germination paper, approximately 2.5 cm from its top edge, with their crease facing the germination paper and embryo facing downward. Seeds were subsequently stratified at 4 °C inside the germination sleeve for at least 3 days. After stratification, germination sleeves were placed randomly in a three-ring binder and kept in a vertical position at room temperature (~22 °C) under long-day conditions (16 h/8 h, day/night) with a light intensity of 120 μmol m^−2^ s^−1^ at the top of the germination sleeve. Seeds that did not initiate germination within 24 h were excluded from the phenotyping process. Phenotypes of 4-day-old wheat seedlings were analyzed by imaging them using a document scanner (Epson, Long Beach, CA). Before imaging, any condensation on the inside of the sheet protector was removed using a Kimwipe (Kimberly-Clark, Irving, TX). Images were acquired as 400 dpi Tagged Image Format (TIF) files.

The scanned seedling images were cropped using the GNU Image Manipulation Program (GIMP) to eliminate scanner and sheet protector edges as well as any non-root tissues. Root traits were assessed from the cropped images using RhizoVision Explorer (Seethepalli et al. 2021). A threshold level of 175 (out of 255) was used on inverted seedling images. Background objects <5 mm were automatically removed to exclude any contribution from image artifacts. Foreground objects (“holes”) <4 mm were also removed automatically. A root pruning threshold of 15 was used to avoid detecting root hairs as lateral roots. Edge smoothing was not enabled. Root numbers were counted manually, and lengths of first true leaves were traced using ImageJ (Abramoff et al. 2004).

### Tissue collection and carotenoid analysis

For carotenoid analysis of seedling tissues, extractions were performed on seedlings grown using the same germination sleeve setup and salt treatment as in the phenotypic analysis, except that five seeds were sown per sleeve. For every treatment, each genotype comprised seeds harvested from five different plants, with seeds from each plant considered as a biological replicate. To obtain a sufficient tissue mass for analysis, 10 seedlings were pooled per biological replicate for the control (0 mM NaCl) treatment and 25 seedlings pooled per biological replicate for the 100 mM NaCl treatment. Given the smaller size of the NaCl-treated seedlings, a larger number of seedlings were pooled per biological replicate to obtain the necessary tissue quantity for carotenoid analysis. Seedlings were dissected manually using a razor blade, with the first-true-leaf and root tissues pooled separately for carotenoid analysis. Samples were immediately flash frozen using liquid nitrogen (N_2_) and stored at −80 °C until analysis.

The frozen tissues were ground into fine powder in liquid N_2_ using a mortar and pestle. For the extraction of carotenoids in first true leaves, 100 mg of sample was used following the previously established method for leaves (Qin et al. 2012; Yu et al. 2022b). Slight modifications were made for the extraction of carotenoids in root tissues due to their generally low amounts in this tissue type. Specifically, a 4:3 ratio (v/v) of acetone to ethyl acetate was used (2.4 mL of acetone: 1.8 mL of ethyl acetate) as the extraction solvent for 450 mg of pooled root sample. A mixture of extraction solvent and water in a 2:3 ratio (v/v) was then used for phase separation. When analyzing the samples using high-performance liquid chromatography (HPLC), the same solvent gradient was applied as outlined previously for both root and leaf samples (Qin et al. 2012).

### Expression analyses for genes involved in carotenoid cleavage

For real-time quantitative PCR (qPCR) analysis, 4-day-old Kronos seedlings grown using the same germination sleeve setup as in the phenotypic analysis were dissected to obtain first-true-leaf and root sections. Root tissues of 4-week-old (tillering) Kronos plants were also collected and used for the real-time qPCR analysis. Total RNA extraction from these tissues was performed using a cetyltrimethylammonium bromide** (**CTAB)-based method (Jaakola et al. 2001), followed by DNase I (Thermo Scientific, Waltham, MA) treatment to remove genomic DNA. First-strand cDNA synthesis was conducted using 3 µg of DNase I-treated total RNA with the iScript Advanced cDNA Synthesis Kit (BioRad, Hercules, CA). Expression levels of the *nine-cis-epoxycarotenoid dioxygenase 3* (*NCED3*), *carotenoid cleavage dioxygenase 7* (*CCD7*), and *carotenoid cleavage dioxygenase 8* (*CCD8*) genes were determined using an ABI 7300 Real-time qPCR system, following a relative standard curve method as previously described (Applied Biosystems 2008; Yu et al. 2022b). For each tissue type, gene expression analysis was performed using three to four biological replicates, each with three technical replicates. Normalization of gene expression in different tissues was carried out using the geometric mean of two reference genes, *Ta2291* and *Ta54227*. The primer sequences used for real-time qPCR analysis are provided in Table S1.

For *in silico* gene expression analysis, transcript levels, measured in transcripts per million, for *CCD7*, *CCD8*, and *NCED3* in different tissues of hexaploid wheat (*Triticum aestivum*) were obtained using the publicly available Wheat Gene Expression Browser (Borrill et al. 2016; Ramírez-González et al. 2018). Specifically, the presented gene expression data were derived from the study on "Developmental time course of Chinese Spring” (Choulet et al. 2014). The coding sequences for wheat *CCD7* and *CCD8* homoeologs (Sigalas et al. 2023) were used as a query for the Gene Expression Browser, including *TaCCD-A7,* TraesCS2A02G414600; *TaCCD-B7*, TraesCS2B02G433800; *TaCCD-D7*, TraesCS2D02G411900; *TaCCD-A8*, TraesCS3A02G274300; *TaCCD-B8*, TraesCS3B02G308000; and *TaCCD-D8*, TraesCS3D02G273500. The *A. thaliana NCED3* mRNA sequence (*At3G14440*) was used to query the RefSeq 1.1 reference sequence, and the best basic local alignment search tool (BLAST®) search result within each wheat subgenome was then used to query the Gene Expression Browser. The accession numbers for the *TaNCED3* homoeologs are: *TaNCED-A3*, TraesCS5A02G374000.1; *TaNCED-B3*, TraesCS5B02G029300.1; and *TaNCED-D3*, TraesCS5D02G038800.1.

### Analysis of the salt-stress response of tillering *hyd* mutant and segregate wild-type plants in a soil growth system

For the segregate wild type and *hyd* mutant lines, seeds were germinated on petri dishes following stratification. The 3-day-old seedlings were transplanted into 0.3-L square pots, which were randomly placed within trays designated as either the control (0 mM NaCl) or salt stress (250 mM NaCl) condition. Each treatment included nine plants per genotype, with three parental lines used for each genotype. Plants were grown in a growth chamber for 3 weeks under long-day conditions (16 h/8 h, day/night) with a daytime light intensity of 100 μmol m^−2^ s^−1^ and a temperature of 22 °C. Before the salt treatment, trays were bottom-watered with a nutrient solution to facilitate establishment. The nutrient solution comprised 0.6 g L^−1^ calcium nitrate, 0.3 g L^−1^ magnesium sulfate, and 0.3 g L^−1^ Grow More 4-18-38 (Grow More Inc., Gardena, CA).

Starting from the 4th week post-planting (tillering stage), plants in the stress condition were flushed with 250 mM NaCl to remove any remaining nutrient solution and then bottom watered with a 250 mM NaCl solution until soil saturation. In contrast, plants in the control treatment group were first flushed with DI water and subsequently received bottom watering with DI water. Trays were also bottom watered with their respective solution/DI water on day 3 and day 6 of the treatment. On day 10 of the treatment, the soil of each tray was flushed with nutrient solution three times, and then bottom watered with nutrient solution to soil saturation. Plants were allowed to recover for 6 days before being harvested. Leaf counts were assessed every 2 days for all plants throughout the salt treatment and until harvest. At harvest, fresh shoot weight, tiller count, and final leaf count were recorded. The dry shoot weight was assessed after drying at 37 °C for 3 days. The shoot water fraction was computed as the proportion of mass lost during drying.

### Analysis of the prolonged salt-stress response of the segregate wild type as well as *hyd-A2hyd-B2* and *hyd-A1hyd-B1hyd-A2hyd-B2* mutant plants in a hydroponic growth system

To examine the response of *hyd-A2 hyd-B2* and *hyd-A1 hyd-B1 hyd-A2 hyd-B2* mutants to prolonged salt stress relative to the segregate wild-type plants, a hydroponic growth system was used in which a Vortex^®^ DC Sprayer (General Hydroponics Inc., Santa Rosa, CA, USA) sprayed nutrient solution directly at the root zone of all plants in each tank. The analysis consisted of 3 tanks, with 18 plants per tank. Ten plants per genotype were used for *hyd-A2 hyd-B2* and *hyd-A1 hyd-B1 hyd-A2 hyd-B2*, while 14 plants were used for the segregate wild-type line.

Seeds were stratified for 4 days at 4 °C, followed by germination at room temperature for 5 days on plates, under a light intensity of 120 μmol m^−2^ s^−1^. The seedlings were then grown in DI water-soaked nylon mesh for 3 days under the same conditions before being planted into a clay pebble growth media (Hydroton^®^; General Hydroponics Inc.) in the hydroponic tanks. Plants were randomly sorted into the tanks to ensure a roughly even distribution of plants per genotype across the tanks. The nutrient solution without added NaCl was supplied to the plants for 2 weeks to allow for establishment in the hydroponic system, after which 50 mM NaCl was added to the nutrient solution. Plants were grown in a growth chamber with a temperature of 22 °C, 56% relative humidity, and long-day settings (16 h/8 h, day/night), at a light intensity of 430 μmol m^−2^ s^−1^. Solutions were replaced every 2 weeks. The counts of surviving tillers were recorded at weeks 1, 2, 3, 5, and 6 after the initiation of the salt treatment. Shoots and roots were harvested at week 6 of the salt treatment, which corresponded roughly to booting or early heading for most plants. Shoot height, shoot fresh mass, and root length were measured at harvest. Shoot and root dry weight were assessed after drying the tissue for 3 days at 37 °C.

### Statistical analysis

For every carotenoid compound within each treatment, means among groups (different genotypes) were compared using one-way analysis of variance (ANOVA) followed by pairwise comparisons between groups using Tukey’s honestly significant difference (HSD) test (*α* = 0.05). Phenotypic parameters were similarly compared among different genotypes. Additionally, for each carotenoid compound within each genotype, comparisons between the salt and the control (0 mM NaCl) treatments were conducted using Student’s *t *test (*α* = 0.05). Statistical analyses were performed using the JMP software (SAS Institute, Cary, NC, USA) as well as using the Agricolae package (Mendiburu and Simon 2015) in RStudio (Posit Software, Boston, MA, USA).

## Results

### Salt-stress conditions, along with a phenotyping procedure for salt-stressed wheat seedlings, were established

To evaluate the suitability of RhizoVision Explorer (Seethepalli et al. 2021), a recently developed image-based analysis tool, for phenotyping salt-stressed wheat seedlings, a pilot study was conducted using images of wheat seedlings obtained from treatments with different salt concentrations (0–150 mM NaCl) (Fig. S2). These seedlings exhibited highly variable root lengths. The results obtained using RhizoVision Explorer showed a close correlation (*R*^2^ = 0.997, slope = 0.989) with manual measurements of root length using ImageJ (Fig. S2a). Additionally, measurements of root length and convex area were well correlated with those measured with GiA Roots, a previously established image analysis tool (Galkovskyi et al. 2012) (Fig. S2b and c). These results confirmed that RhizoVision Explorer reliably identifies and measures roots in images generated in our experimental setup, substantiating its use in our subsequent phenotyping analysis of salt-stressed *hyd* mutant and segregate wild-type wheat seedlings.

To determine an appropriate salt concentration that could elicit a discernable phenotypic response in wheat seedlings without drastically impeding growth, seed germination rates and seedling phenotypes were initially assessed in a small set of segregate wild-type, *hyd-A1 hyd-B1*, and *hyd-A2 hyd-B2* seeds under varying salt treatments (0, 50, 100, 150, and 200 mM NaCl). A salt tolerance index (STI), defined as the ratio of the mean of a given trait with and without salt treatment (Wu et al. 2019), was established for the length of the first true leaf, root length, and root network area (area of root pixels in the image) (Table S2). These results indicated that a concentration of 100 mM NaCl, with STI values ranging from 0.20 to 0.44 for root length, caused notably stunted seedling growth while still allowing for image-based phenotyping and the collection of a sufficient amount of tissue for biochemical analysis (Table S2).

To assess the possibility of salt stress occurring before germination (e.g., during imbibition), seeds of Kronos, the parental line of *hyd* mutants and segregate wild type, were imbibed in salt solutions or DI water prior to applying various salt treatments. No differences in root length or length of the first true leaf were observed among seeds that were imbibed in different salt solutions (0, 100, 200 mM NaCl) and continued with the salt treatment compared to those imbibed in DI water and later transferred to salt solutions (Fig. S3a and b). Similarly, there were no noticeable differences in growth among seedlings that were imbibed in either a 200 mM NaCl solution or in DI water and then germinated in DI water (Fig. S3c–e). These results suggest that salt stress applied at imbibition does not appear to affect wheat seedling growth. As such, we focused on evaluating differences in salt-stress response of the *hyd* mutant and segregate wild-type lines during or after germination in this study.

### Mutant seedlings with defective HYD2 homoeologs exhibited longer first true leaves and a higher root count compared to segregate wild-type seedlings when subjected to salt stress

**Fig. 1:**
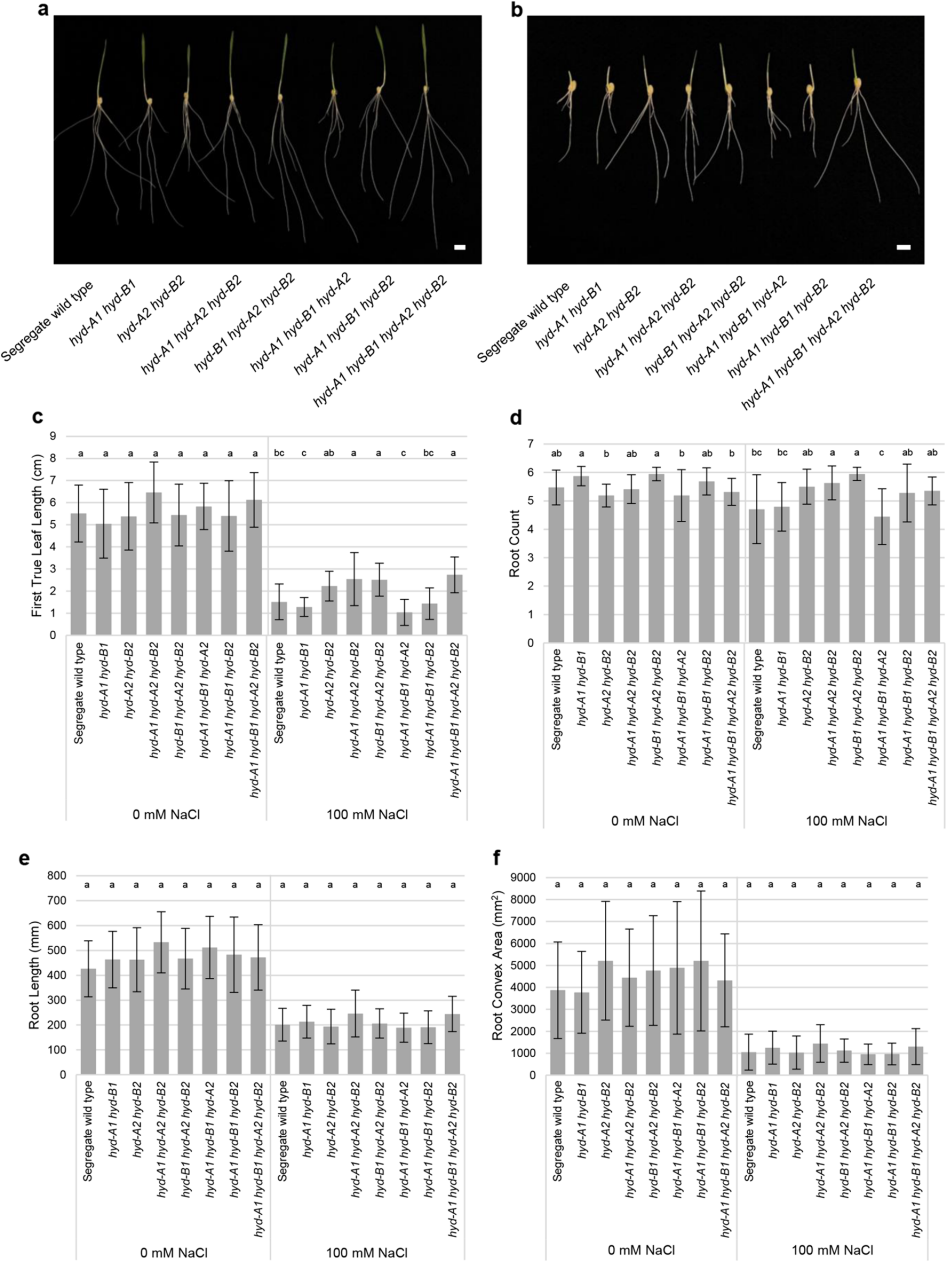
Growth of *hyd* mutant and segregate wild-type seedlings subjected to control (0 mM NaCl) and salt-stress treatments. Representative seedlings for each genotype are shown for the 0 mM NaCl treatment (**a**) and the salt-stress treatment (100 mM NaCl) (**b**). Effects of salt stress on first-true-leaf length (**c**), root count (**d**), root length (**e**), and root convex area (**f**) are shown. *Error bars* represent ± standard deviation (*n* = 20). Statistically significant differences (*p* < 0.05) between groups within a treatment are denoted with *different letters*

To understand whether seedlings of various *hyd* mutants may respond differently to salt stress, seedling phenotypes of the segregate wild type and *hyd* mutant lines were examined after growth in either DI water or 100 mM NaCl. Under the control condition (i.e., treatment with DI water), no significant differences were observed among the *hyd* mutant and segregate wild-type lines for all assessed traits, including first-true-leaf length, root length, root count, and root convex area (Fig. 1a and c–f), suggesting normal germination and growth in *hyd* mutants compared to the segregate wild-type line in the absence of salt stress. However, in the presence of salt stress, all genotypes (including the segregate wild-type line) exhibited smaller root convex area, shorter root and first-true-leaf lengths, and similar or reduced root count compared to those germinated in DI water (Fig. 1b–f). Under the salt treatment, an intriguing observation was that mutant seedlings lacking functional HYD2, including *hyd-A2 hyd-B2*, *hyd-A1 hyd-A2 hyd-B2*, *hyd-B1 hyd-A2 hyd-B2*, and *hyd-A1 hyd-B1 hyd-A2 hyd-B2*, showed longer first-true-leaf length relative to seedlings of the segregate wild-type line (ranging from a 32% increase in length in *hyd-A2 hyd-B2* to a 45% increase in *hyd-A1 hyd-B1 hyd-A2 hyd-B2*) (Fig. 1b and c). A slight but significantly higher root count was also observed in *hyd-A1 hyd-A2 hyd-B2* and *hyd-B1 hyd-A2 hyd-B2* (Fig. 1b and d), while all *hyd* mutants and segregate wild-type lines did not differ in root length and convex areas of the salt-stressed root systems (Fig. 1e and f). Notably, mutant lines lacking functional HYD2 also exhibited significantly higher STIs than the segregate wild type for first-true-leaf length and root count (Table S3). These results collectively suggest a role of HYD2 in the response of wheat seedlings to salt stress.

### Neoxanthin and violaxanthin concentrations were reduced in roots of mutant seedlings with lesions in *HYD2 *homoeologs

To assess carotenoid levels in *hyd* mutant and segregate wild-type lines and understand their relation to differential growth responses to salt stress, carotenoid profiles were analyzed for mutants and the segregate wild type in first true leaves and roots under control (0 mM NaCl) and salt-stress (100 mM NaCl) conditions (Tables 1 and 2). In seedlings treated with 0 mM NaCl, β-xanthophylls (neoxanthin and violaxanthin) were reduced in first true leaves of most *hyd* mutants, with the most pronounced reduction observed in mutants lacking functional HYD1 homoeologs (Table 1). On the other hand, increases in β-carotene were observed in combinatorial mutants containing *hyd-A1 hyd-B1*, likely due to a decrease in carotenoid β-hydroxylation activities, which prevents the conversion of β-carotene to downstream β-xanthophylls and leads to a buildup of β-carotene in these mutants (Fig. S1; Table 1). Additionally, increases in lutein accumulation were only observed in *hyd-A1 hyd-B1* and *hyd-A1 hyd-B1 hyd-A2 hyd-B2* relative to the segregate wild type (Table 1). The concentrations of total carotenoids did not differ significantly among *hyd* mutants and the segregate wild type (Table 1). In salt-stressed seedlings, the relative accumulation for individual and total carotenoids between mutants and the segregate wild type largely resembled those found in seedlings treated with 0 mM NaCl (Table 1). For each genotype, significant (*p* < 0.01 or *p* < 0.05) increases in carotenoid concentrations were observed in first true leaves upon salt stress: including neoxanthin in the segregate wild type and *hyd-B1 hyd-A2 hyd-B2*, violaxanthin in *hyd-B1 hyd-A2 hyd-B2* and *hyd-A1 hyd-B1 hyd-B2*, lutein in the segregate wild type, *hyd-A1 hyd-B1*, *hyd-B1 hyd-A2 hyd-B2*, *hyd-A1 hyd-B1 hyd-A2*, and *hyd-A1 hyd-B1 hyd-B2*, β-carotene in *hyd-A1 hyd-B1 hyd-A2*, and total carotenoids in *hyd-B1 hyd-A2 hyd-B2* and *hyd-A1 hyd-B1 hyd-B2* (Table 1).

**Table 1:** Carotenoid concentrations (mmol mol^−1^ chlorophylls *a* + *b*) in first true leaves of *hyd* mutant and segregate wild-type seedlings germinated under 0 or 100 mM of NaCl

Genotype	Neoxanthin		Violaxanthin		Lutein		β-Carotene		Total carotenoids	
	0 mM NaCl	100 mM NaCl	0 mM NaCl	100 mM NaCl	0 mM NaCl	100 mM NaCl	0 mM NaCl	100 mM NaCl	0 mM NaCl	100 mM NaCl
Segregate Wild type	19.93 ± 0.95^a^	21.92 ± 1.58^a^ (*)	30.75 ± 7.93^a^	40.15 ± 11.19^a^	98.34 ± 8.88^c^	123.21 ± 20.33^a^ (*)	39.13 ± 2.14^d^	43.42 ± 4.32^bc^	188.15 ± 18.77^ab^	228.69 ± 36.36^a^
*hyd-A1* *hyd-B1*	13.69 ± 1.37^c^	12.67 ± 1.31^c^	10.14 ± 1.32^c^	13.74 ± 4.19^ cd^	113.57 ± 5.20^ab^	137.78 ± 22.53^b^ (*)	45.36 ± 2.40^bc^	50.09 ± 7.05^abc^	182.76 ± 9.45^ab^	214.29 ± 34.90^a^
*hyd-A2* *hyd-B2*	17.51 ± 1.06^b^	18.72 ± 2.02^b^	33.67 ± 5.74^a^	29.41 ± 4.24^ab^	101.03 ± 6.64^bc^	100.23 ± 8.51^ab^	42.90 ± 1.56^bcd^	41.04 ± 2.93^c^	195.12 ± 11.98^a^	189.40 ± 17.26^a^
*hyd-A1* *hyd-A2* *hyd-B2*	17.24 ± 0.41^b^	18.11 ± 1.87^b^	21.62 ± 2.59^b^	26.79 ± 8.10^b^	101.54 ± 6.76^bc^	123.76 ± 28.40^ab^	45.18 ± 2.01^bc^	47.59 ± 5.21^bc^	185.58 ± 10.61^ab^	216.25 ± 42.38^a^
*hyd-B1* *hyd-A2* *hyd-B2*	17.88 ± 0.35^ab^	19.64 ± 0.58^ab^ (**)	20.75 ± 1.81^b^	24.95 ± 2.94^bc^ (*)	96.59 ± 3.33^c^	110.43 ± 5.14^ab^ (**)	39.95 ± 1.52^ cd^	41.15 ± 0.67^c^	175.16 ± 4.78^ab^	196.17 ± 8.83^a^ (**)
*hyd-A1* *hyd-B1* *hyd-A2*	9.00 ± 1.36^d^	7.75 ± 0.98^d^	7.45 ± 1.25^ cd^	6.99 ± 1.75^de^	107.35 ± 8.63^abc^	129.68 ± 19.62^ab^ (*)	45.76 ± 2.86^b^	49.36 ± 5.74^abc^ (**)	169.56 ± 13.22^b^	193.78 ± 26.50^a^
*hyd-A1* *hyd-B1* *hyd-B2*	7.56 ± 1.57^d^	8.02 ± 0.23^d^	5.09 ± 0.98^ cd^	7.42 ± 1.21^de^ (*)	111.67 ± 8.65^abc^	136.97 ± 11.82^ab^ (*)	48.43 ± 4.35^b^	53.08 ± 5.16^ab^	172.75 ± 14.52^ab^	205.49 ± 17.99^a^ (*)
*hyd-A1* *hyd-B1* *hyd-A2* *hyd-B2*	0.45 ± 0.62^e^	ND	ND	ND	118.94 ± 8.17^a^	132.26 ± 12.01^ab^	54.97 ± 3.53^a^	58.18 ± 5.27^a^	174.36 ± 11.23^ab^	190.44 ± 17.08^a^

Values displayed are means ± standard deviation of four to six biological replicates. Statistically significant differences (*p* < 0.05) within each column are denoted with different letters. For individual carotenoid molecule or total carotenoids of each genotype, statistically significant differences between the 0 mM NaCl and 100 mM NaCl treatments are indicated with *(*p* < 0.05) and **(*p* < 0.01), respectively

*ND* not detected

**Table 2:** Carotenoid concentrations (nmol g^−1^ fresh tissue) in roots of *hyd* mutant and segregate wild-type seedlings germinated under 0 or 100 mM of NaCl

Genotype	Neoxanthin		Violaxanthin		β-Carotene		Total carotenoids	
	0 mM NaCl	100 mM NaCl	0 mM NaCl	100 mM NaCl	0 mM NaCl	100 mM NaCl	0 mM NaCl	100 mM NaCl
Segregate Wild type	0.33 ± 0.01^a^	0.29 ± 0.04^a^	1.59 ± 0.34^a^	1.47 ± 0.25^a^	0.43 ± 0.16^b^	0.38 ± 0.12^b^	2.35 ± 0.42^a^	1.85 ± 0.23^a^ (*)
*hyd-A1* *hyd-B1*	0.30 ± 0.03^ab^	0.28 ± 0.04^ab^	1.43 ± 0.13^ab^	1.41 ± 0.08^a^	0.46 ± 0.19^b^	0.44 ± 0.09^ab^	2.19 ± 0.32^a^	1.85 ± 0.14^a^
*hyd-A2* *hyd-B2*	ND	ND	0.26 ± 0.05^d^	0.23 ± 0.03^c^	0.42 ± 0.04^b^	0.37 ± 0.03^b^	0.68 ± 0.07^c^	0.60 ± 0.04^c^ (*)
*hyd-A1* *hyd-A2* *hyd-B2*	ND	ND	0.16 ± 0.04^d^	0.15 ± 0.04^ cd^	0.49 ± 0.07^ab^	0.51 ± 0.16^ab^	0.65 ± 0.11^c^	0.51 ± 0.16^c^
*hyd-B1* *hyd-A2* *hyd-B2*	ND	ND	0.21 ± 0.04^d^	0.19 ± 0.04^ cd^	0.51 ± 0.06^ab^	0.43 ± 0.03^ab^ (*)	0.72 ± 0.10^c^	0.43 ± 0.03^c^ (**)
*hyd-A1* *hyd-B1* *hyd-A2*	0.24 ± 0.04^b^	0.22 ± 0.06^b^	0.79 ± 0.04^c^	0.96 ± 0.13^b^ (*)	0.44 ± 0.07^b^	0.41 ± 0.06^ab^	1.47 ± 0.11^b^	1.37 ± 0.18^b^
*hyd-A1* *hyd-B1* *hyd-B2*	0.32 ± 0.08^a^	0.26 ± 0.01^ab^	1.15 ± 0.17^b^	1.12 ± 0.05^b^	0.45 ± 0.09^b^	0.45 ± 0.03^ab^	1.93 ± 0.33^ab^	1.56 ± 0.09^ab^
*hyd-A1* *hyd-B1* *hyd-A2* *hyd-B2*	ND	ND	ND	ND	0.72 ± 0.15^a^	0.60 ± 0.12^a^	0.72 ± 0.15^c^	0.60 ± 0.12^c^

Values displayed are means ± standard deviation of four to six biological replicates. Statistically significant differences (*p* < 0.05) within each column are denoted with different letters. For individual carotenoid molecule or total carotenoids of each genotype, statistically significant differences between the 0 mM NaCl and 100 mM NaCl treatments are indicated with *(*p* < 0.05) and **(*p* < 0.01), respectively

*ND* not detected

Interesting patterns related to *hyd2* mutations emerged when carotenoid profiles of seedling roots were analyzed (Table 2). The primary carotenoid compounds accumulated in this tissue were neoxanthin, violaxanthin, and β-carotene, though their levels (in the range of nmol g^−1^) were considerably lower than those in first true leaves (in the range of mmol per mol chlorophylls *a* + *b*) (Tables 1 and 2). In seedlings treated with 0 mM NaCl, mutants lacking functional HYD2 showed greatly reduced concentrations of β-xanthophylls, specifically 83–100% reductions (and undetectable in *hyd-A1 hyd-B1 hyd-A2 hyd-B2*) in violaxanthin and undetectable levels of neoxanthin. β-Carotene concentrations showed a notable 68% increase in *hyd-A1 hyd-B1 hyd-A2 hyd-B2* (Table 2). Total carotenoids decreased by ~70% in mutants containing *hyd-A2 hyd-B2*. Interestingly, β-carotene was the only detectable carotenoid in seedlings roots of *hyd-A1 hyd-B1 hyd-A2 hyd-B2*. In salt-stressed seedlings, the relative accumulation of both individual and total carotenoids between mutants and the segregate wild type closely mirrored that observed in seedlings treated with 0 mM NaCl (Table 2). When carotenoid concentrations in the presence and absence of salt stress were compared for each genotype, the most notable changes were an increase in violaxanthin in *hyd-A1 hyd-B1 hyd-A2* and a decrease in β-carotene and total carotenoids in *hyd-B1 hyd-A2 hyd-B2* (Table 2).

### Genes responsible for converting β-carotenoids into ABA and SLs were undetectable by real-time qPCR in tetraploid wheat seedling roots

**Fig. 2:**
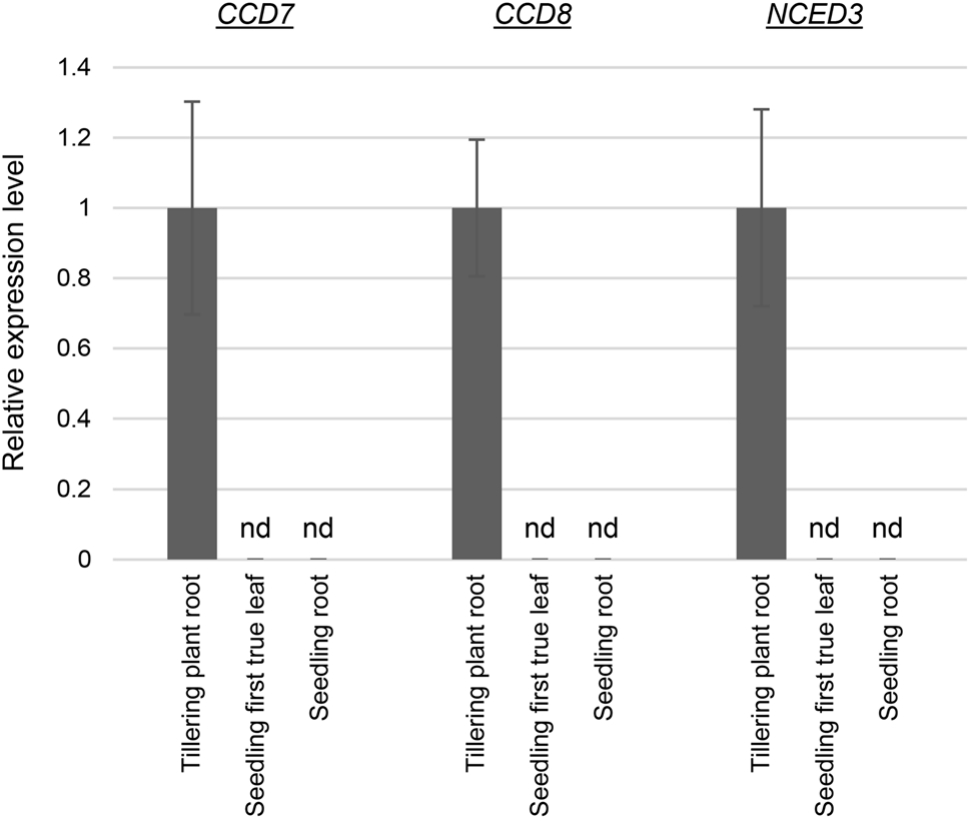
Relative expression of candidate carotenoid cleavage genes in tetraploid wheat tissues. Transcript quantification was carried out using the relative standard curve method (Applied Biosystems 2008), with gene expression normalized using the geometric mean of two reference genes, *Ta2291* and *Ta54227*. Values shown are the mean ± SD of 3–4 biological replicates. CCD, carotenoid cleavage dioxygenase; NCED, nine-*cis*-epoxycarotenoid dioxygenase. Expression of *CCD7*, *CCD8*, and *NCED3* were below the limit of reliable detection by real-time qPCR (nd) in first true leaves and roots of 4-day-old seedlings

To explore possible relationships between changes in seedling root carotenoid profiles, particularly decreased β-xanthophylls and increased β-carotene (collectively termed β-carotenoids), and the differential growth phenotypes in mutant seedlings containing *hyd2* mutations, the expression patterns of genes encoding cleavage enzymes responsible for producing carotenoid-derived phytohormones, ABA and SLs, were examined using real-time qPCR (Fig. 2). Transcripts of *CCD7* and *CCD8*, involved in the synthesis of SLs from β-carotene, and *NCED3*, involved in the synthesis of ABA from β-xanthophylls, were detected in the roots of tillering (4-week-old) tetraploid wheat plants (Fig. 2), However, they were below the limit for reliable detection in roots and first true leaves of 4-day-old seedlings (Fig. 2).

The expression levels of these genes were also assessed in hexaploid wheat using publicly available transcriptome data (Ramírez-González et al. 2018) (Fig. S4). The subgenome homoeologs of both *CCD7* and *CCD8* were below or slightly above the detection thresholds in seedling shoots in transcriptome analysis (Fig. S4a and b). Relatively higher expression was observed in vegetative tissues and spikes for *CCD7* homoeologs, and more transcripts accumulated in roots and spikes of mature plants for *CCD8* homoeologs (Fig. S4a and b). Similar to *CCD7* and *CCD8* homoeologs, the expression levels of *NCED3* homoeologs are generally lower in seedling roots and shoots relative to those found in tissues of mature plants (Fig. S4c).

### Tillering mutant plants with defective HYD2 homoeologs showed similar growth traits to segregate wild-type plants under salt stress, except for compromised growth in *hyd-A1hyd-B1hyd-A2hyd-B2*

**Fig. 3:**
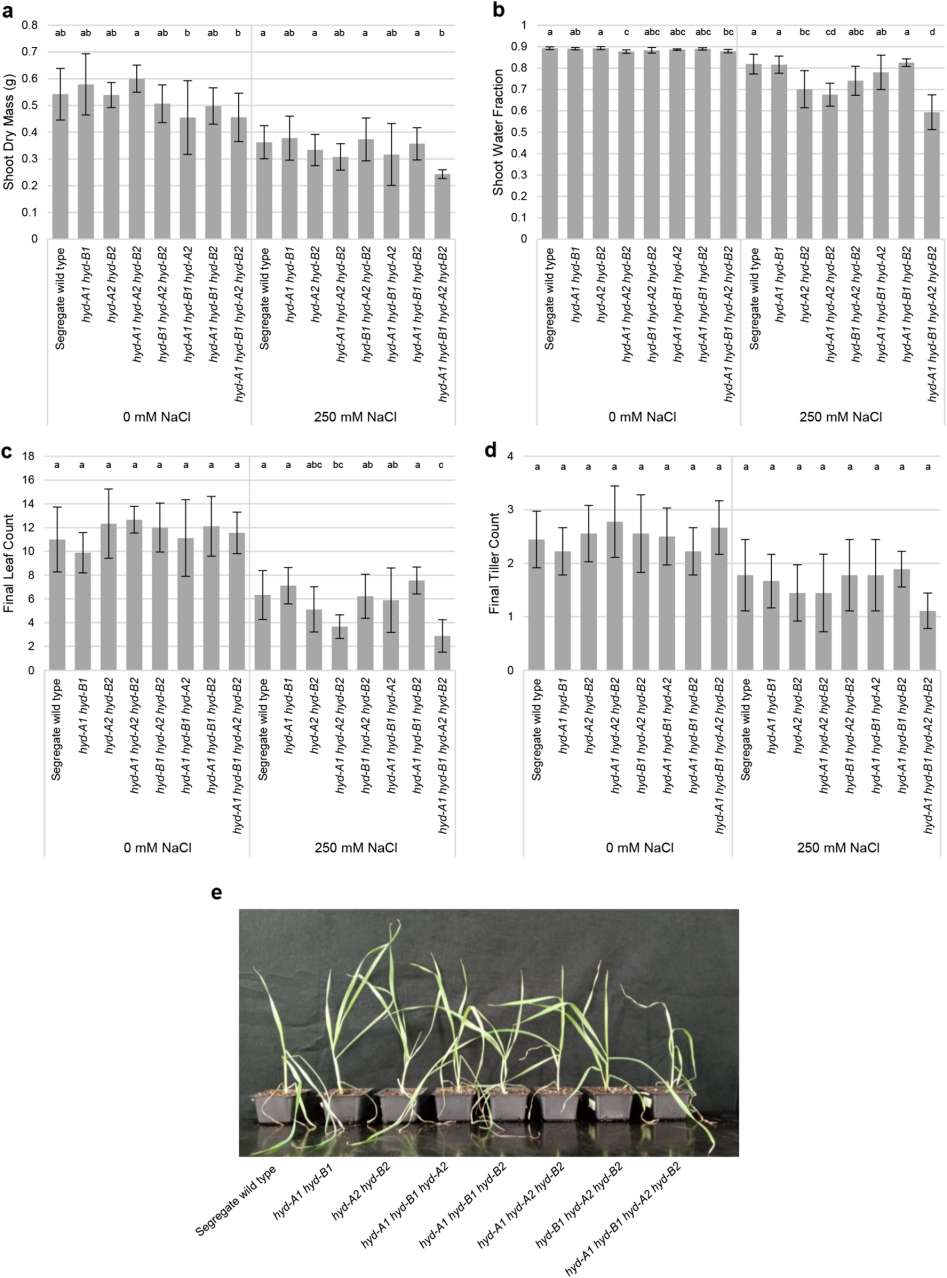
Growth of tillering *hyd* mutant and segregate wild-type plants subjected to control (0 mM NaCl) and salt-stress treatments. Salt stress was applied at 250 mM NaCl, followed by recovery in a soil-based system. Phenotypic values after harvest for shoot dry mass (**a**), shoot water fraction (**b**), leaf count (**c**), and tiller count (**d**) are shown. Representative plants for each genotype are shown in (**e**) (note that the genotypes are ordered differently from panels **a**–**d**). *Error bars* represent ± standard deviation (*n* = 9). Statistically significant differences (*p* < 0.05) between groups within a treatment are denoted with *different letters*

To investigate how *hyd* mutant plants respond to salt stress beyond the seedling stage, 4-week-old (tillering) mutant and segregate wild-type plants grown in soil were subjected to a salt treatment of 250 mM NaCl, followed by a recovery period. A salt concentration higher than that used for seedlings (100 mM) was employed to elicit a discernable effect on growth in tillering plants, as wheat plants at more advanced growth stages generally exhibit greater tolerance to salt stress than seedlings (Maas and Poss 1989). No significant differences in shoot dry mass, shoot water fraction, leaf count, and tiller count were observed for segregate wild type and *hyd2* mutant plants under the control (0 mM NaCl) condition (Fig. 3 and Fig. S5a). Throughout salt-stress treatment and recovery, most mutant lines displayed similar numbers of healthy leaves per individual plant relative to segregate wild-type plants, except for *hyd-A1 hyd-A2 hyd-B2* and *hyd-A1 hyd-B1 hyd-A2 hyd-B2*, which showed poor growth even during the recovery phase (Fig. 3c, e and Fig. S5b). In particular, the *hyd-A1 hyd-B1 hyd-A2 hyd-B2* mutant was severely impacted by the salt treatment, showing significantly lower shoot dry mass (33% reduction) and shoot water fraction (i.e., fresh shoot mass/dry shoot mass) (28% reduction) at the end of the salt treatment relative to the segregate wild type (Fig. 3a and b). Additionally, *hyd-A2 hyd-B2* and *hyd-A1 hyd-A2 hyd-B2* also exhibited decreased shoot water fraction, though the reduction was less pronounced than *hyd-A1 hyd-B1 hyd-A2 hyd-B2* (Fig. 3b).

**Fig. 4:**
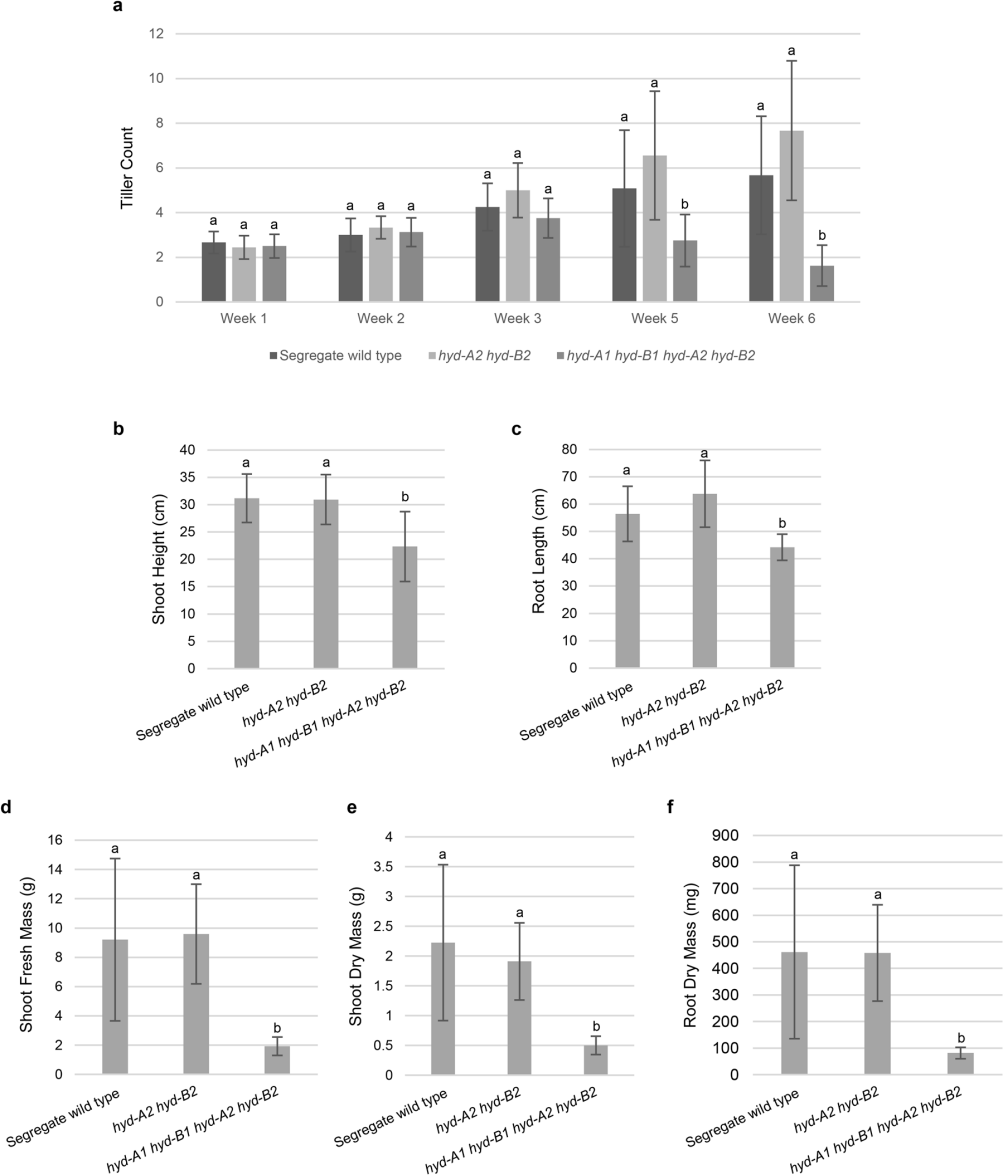
Growth of tillering *hyd* mutant and segregate wild-type plants subjected to a prolonged salt stress at 50 mM NaCl in a hydroponic system. Tiller counts were determined over the course of growth (**a**). Statistically significant differences (*p* < 0.05) in tiller count among the genotypes at each time point are indicated with different letters. Phenotypic values at harvest for shoot height (**b**), root length (**c**), and shoot fresh mass (**d**) are shown. Shoot dry mass (**e**) and root dry mass (**f**) after drying are also shown. *Error bars* represent ± standard deviation (*n* = 14 for segregate wild type; *n* = 10 for *hyd-A2 hyd-B2* and *hyd-A1 hyd-B1 hyd-A2 hyd-B2*). Statistically significant differences (*p* < 0.05) between groups are denoted with *different letters*

To examine how *hyd* mutant plants respond to prolonged but less severe salt stress, the *hyd-A2 hyd-B2* and *hyd-A1 hyd-B1 hyd-A2 hyd-B2* mutants as well as segregate wild-type plants were subjected to a 6-week salt treatment using 50 mM NaCl, starting with 3-week-old plants (Fig. 4). This experiment was conducted with a hydroponic growth system to ensure consistent control of nutrient and salt supply. The *hyd-A2 hyd-B2* mutant plants performed similarly to the segregate wild-type plants over the course of the salt treatment (Fig. 4). By contrast, the *hyd-A1 hyd-B1 hyd-A2 hyd-B2* mutant plants displayed a decline in tiller count during the salt treatment (Fig. 4a). Additionally, the *hyd-A1 hyd-B1 hyd-A2 hyd-B2* mutant also exhibited poor growth under prolonged salt stress, with reduced shoot height and root length alongside greater than 50% reductions in shoot and root biomass at the end of the salt treatment (Fig. 4b–f).

## Discussion

Our previous analysis showed that tetraploid wheat *hyd* mutants carrying lesions in *HYD1* and *HYD2* homoeologs possess diverse carotenoid compositions in leaf and grain tissues (Bekkering et al. 2023). When not subjected to stresses, these mutants also displayed growth patterns similar to those of the segregate wild type, with only minor developmental changes observed in the quadruple mutant *hyd-A1 hyd-B1 hyd-A2 hyd-B2* (Bekkering et al. 2023). In this study, we utilized combinatorial *hyd* mutants with differing carotenoid concentrations to investigate the potential role of carotenoids in the response of wheat plants to salt stress, at the seedling and tillering growth stages. Interestingly, we observed improved growth, particularly longer first true leaves, under salt stress in all mutant seedlings lacking functional HYD2 compared to the segregate wild type, including the *hyd-A2 hyd-B2*, *hyd-A1 hyd-A2 hyd-B2*, *hyd-B1 hyd-A2 hyd-B2*, and *hyd-A1 hyd-B1 hyd-A2 hyd-B2* mutant lines (Fig. 1). In these mutant seedlings lacking functional HYD2, a substantial reduction or even elimination of β-xanthophylls in the roots, accompanied by a slight enrichment of β-carotene, was also observed (Table 2). These results collectively suggest a potential connection between the carotenoid levels in the seedling roots and their response to salt stress in these mutants.

Plants employ a combination of tolerance and avoidance strategies to respond to high-salt conditions in the environment. Salt-stress tolerance is often indicated by relative changes in tissue-specific growth rates and biomass production under saline conditions (van Zelm et al. 2020). Additionally, plants adapt to elevated salt levels through avoidance strategies, such as modifying the gravitropic response to direct root growth away from high-salt areas (Galvan-Ampudia et al. 2013; Yu et al. 2022a; Zou et al. 2022) and altering root system architecture to reduce the initiation and elongation of lateral roots and root hairs under salt stress (Zou et al. 2022). The increased growth of first true leaves in *hyd2* mutant seedlings compared to segregate wild-type seedlings observed in this study indicates an enhanced salt-stress response at this developmental stage resulting from loss-of-function *hyd2* mutations. However, additional research is needed to elucidate the stress tolerance and avoidance mechanisms at play in these seedlings.

The β-xanthophyll-derived phytohormone ABA reportedly plays a critical role in regulating salt-stress responses in plants (Vishwakarma et al. 2017; Yu et al. 2020). Recent studies have also highlighted the involvement of SLs, a class of β-carotene-derived phytohormone, in salt-stress response; they promote physiological responses that enhance salt tolerance in a manner overlapping but not entirely redundant with ABA (Korek and Marzec 2023). Transcripts of *CCD7*, *CCD8*, and *NCED3*, encoding cleavage enzymes for ABA and SL biosyntheses, were below the limit of reliable detection for real-time qPCR in 4-day-old tetraploid wheat seedling roots and first true leaves (Fig. 2). On the other hand, publicly available transcriptome data for hexaploid wheat showed low but detectable expression of these genes in seedling roots (Fig. S4) (Choulet et al. 2014; Ramírez-González et al. 2018). Despite the low or undetectable levels of these carotenoid cleavage genes, it remains possible that ABA and/or SL levels, as well as their respective signaling pathways, may undergo modifications in *hyd2* mutant seedlings, affecting their response to salt stress and growth phenotypes. Quantification of ABA and SLs in the *hyd2* mutant and segregate wild-type seedlings could shed lights on the connection between modified carotenoid profiles in *hyd2* mutants and their responses to salt stress.

Another interesting observation of this study was the distinct growth patterns exhibited by seedlings lacking functional homoeologs of HYD2, as compared to more established tillering plants facing high-salt concentrations (Figs. 1, 3, and 4; Fig. S5b). This prompts questions about the mechanisms that govern growth stage-specific responses of wheat plants to salt stress. Since altered carotenoid profiles in *hyd2* mutant seedlings are implicated in their differential salt responses relative to segregate wild-type seedlings, future investigations could examine the expression patterns of genes associated with carotenoid metabolism, extending beyond *NCED3*, *CCD7*, and *CCD8*, in both seedling and tillering plants exposed to elevated salt concentrations.

Our previous investigation focusing on provitamin A biofortification of wheat grains revealed that blocking the reactions catalyzed by HYD2 in the *hyd-A2 hyd-B2* mutant led to a twofold increase in β-carotene (provitamin A carotenoid) levels in mature grains, with no adverse effects on plant growth (Yu et al. 2022b). An exciting finding from this study was that seedling and tillering plants of the *hyd-A2 hyd-B2* mutant performed similarly to segregate wild-type plants when subjected to acute and prolonged salt stress, suggesting that these provitamin A biofortified plants did not sustain any penalty for salt-stress response. On the other hand, the tillering *hyd-A1 hyd-B1 hyd-A2 hyd-B2* quadruple mutant displayed high susceptibility to salt stress, especially with prolonged exposure in the hydroponic growth system (Figs. 3 and 4). This increased susceptibility in the quadruple mutant could be attributed, at least in part, to reduced ABA biosynthesis resulting from decreased production of β-xanthophyll precursors, as suggested in our previous study (Bekkering et al. 2023).

## Conclusions

This study revealed an interesting and unexpected enhancement in the response to salt stress in tetraploid wheat mutant seedlings with reduced β-xanthophylls and elevated levels of β-carotene in their roots. In doing so, it fills a previously unaddressed gap in our understanding of the relationship between salinity response and carotenoid concentrations during the initial growth stages of wheat plants. Furthermore, it demonstrated that the metabolic strategy used to increase grain β-carotene content in the *hyd-A2 hyd-B2* double mutant of tetraploid wheat did not compromise the salt-stress response in seedling and tillering plants.

### Supplementary Information

Below is the link to the electronic supplementary material.

Supplementary file1 A simplified carotenoid biosynthetic pathway in wheat. The HYD-catalyzed reactions are indicated in bold. Dashed arrows denote multiple reactions. PSY, phytoene synthase; PDS, phytoene desaturase; ZISO, *ζ*-carotene isomerase; ZDS, *ζ*-carotene desaturase; CRTISO, carotenoid isomerase; LCYb, lycopene *β*-cyclase; LCYe, lycopene *ε*-cyclase; HYD, carotenoid *β*-hydroxylase (non-heme di-iron type); CYP, cytochrome P450 type carotenoid hydroxylase; ZEP, zeaxanthin epoxidase; VDE, violaxanthin de-epoxidase; NXS, neoxanthin synthase; CCD, carotenoid cleavage dioxygenase; D27, DWARF27 (*β*-carotene isomerase); NCED, nine-*cis*-epoxycarotenoid dioxygenase (PDF 102 KB)

Supplementary file2 Implementation of RhizoVision Explorer for phenotyping salt-stressed wheat seedlings. (a) Comparison of root lengths measured using RhizoVision Explorer with “ground truth” manual measurements on seedlings of diverse lengths and root angles. Comparison of root length (b) and convex area (c) outputs from RhizoVision Explorer with those of GiA Roots. Adaptive image thresholding was used in GiA Roots to capture as much of the root system as possible (PDF 20 KB)

Supplementary file3 Growth of seedlings when seeds were imbibed in deionized water or salt solutions. First true leaf lengths (a) and root lengths (b) when imbibed with or without salt stress [in the salt solution-soaked germination paper or in deionized (DI) water, respectively; n = 10 per treatment]. Effects of salt stress during imbibition on seeds germinating in DI water are presented for the first-true-leaf lengths (c), root lengths (d), and root count (e) (n = 20 per treatment). Error bars represent ± standard deviation. No statistically significant differences were detected within treatments (PDF 30 KB)

Supplementary file4 *In silico* analysis of candidate carotenoid cleavage gene expression in hexaploid wheat across different tissue types and developmental stages. Transcript levels, in transcripts per million, are shown for the homoeologs of *TaCCD7* (a), *TaCCD8* (b), and *TaNCED3* (c). CCD, carotenoid cleavage dioxygenase; NCED, nine-*cis*-epoxycarotenoid dioxygenase. The presented gene expression data were derived from the study on “Developmental time course of Chinese Spring” (Choulet et al. 2014). The variable “n” denotes the number of RNAseq libraries constructed and sequenced for each specific wheat tissue (PDF 143 KB)

Supplementary file5 Growth of tillering *hyd* mutant and segregate wild-type plants subjected to control (0 mM NaCl) and salt-stress treatments. Salt stress was applied at 250 mM NaCl followed by recovery in a soil-based system. Leaf counts of plants under the unstressed (0 mM NaCl) (a) and acute salt stress (250 mM NaCl) (b) treatments are shown. Error bars represent ± standard deviation (n = 9). Initiation of recovery on day 10 of the salt treatment is also denoted (PDF 348 KB)

Supplementary file6 (DOCX 44 KB)

## Data Availability

The datasets generated and/or analyzed during the current study are available from the corresponding author on reasonable request.

## Abbreviations

ABA: Abscisic acid
ANOVA: Analysis of variance
BLAST: Basic local alignment search tool
CCD: Carotenoid cleavage dioxygenase
CTAB: Cetyltrimethylammonium bromide
DI: Deionized
GIMP: GNU Image Manipulation Program
HPLC: High-performance liquid chromatography
HSD: Honestly significant difference
HYD: Carotenoid β-hydroxylase
LCYe: Lycopene ε-cyclase
MAX: More axillary growth
N_2_: Nitrogen
NCED: Nine-*cis*-epoxycarotenoid dioxygenase
Or: Orange
qPCR: Quantitative PCR
RNAi: RNA interference
ROS: Reactive oxygen species
SL: Strigolactone
STI: Salt tolerance index
TIF: Tagged image format
