# Supplementary figures and images for: Distinct growth patterns in seedling and tillering wheat plants suggests a developmentally restricted role of HYD2 in salt-stress response
Source: Plant Cell Rep. 2024 Apr 17;43(5):119. doi: 10.1007/s00299-024-03206-x (PMC11024023; doi:10.1007/s00299-024-03206-x)

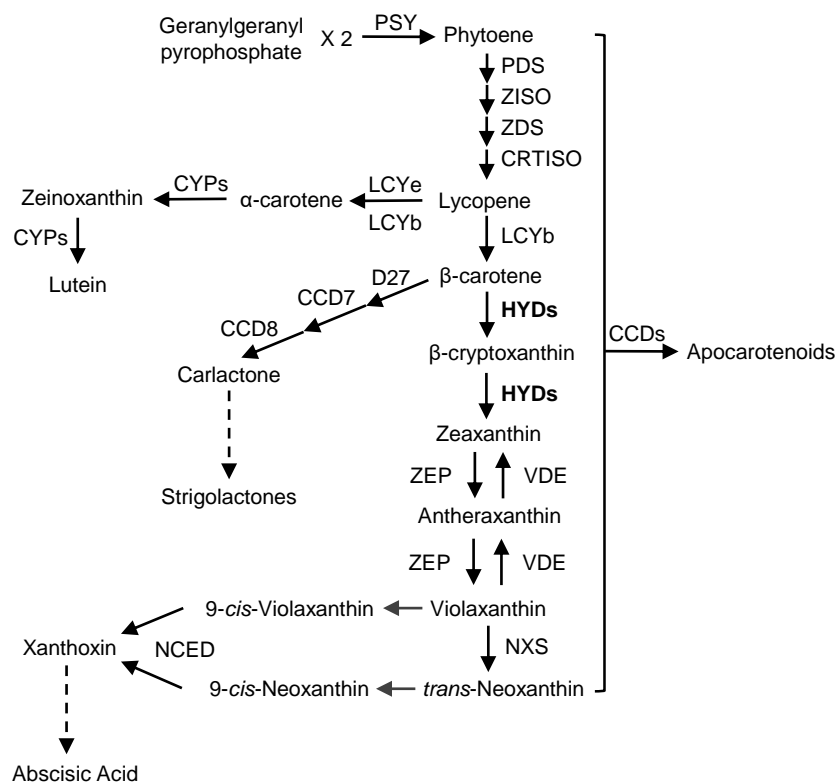

Supplement: Supplementary file 1 — Supplementary file1 A simplified carotenoid biosynthetic pathway in wheat. The HYD-catalyzed reactions are indicated in bold. Dashed arrows denote multiple reactions. PSY, phytoene synthase; PDS, phytoene desaturase; ZISO, ζ-carotene isomerase; ZDS, ζ-carotene desaturase; CRTISO, carotenoid isomerase; LCYb, lycopene β-cyclase; LCYe, lycopene ε-cyclase; HYD, carotenoid β-hydroxylase (non-heme di-iron type); CYP, cytochrome P450 type carotenoid hydroxylase; ZEP, zeaxanthin epoxidase; VDE, violaxanthin de-epoxidase; NXS, neoxanthin synthase; CCD, carotenoid cleavage dioxygenase; D27, DWARF27 (β-carotene isomerase); NCED, nine-cis-epoxycarotenoid dioxygenase (PDF 102 KB) [file 299_2024_3206_MOESM1_ESM.pdf]

**a**

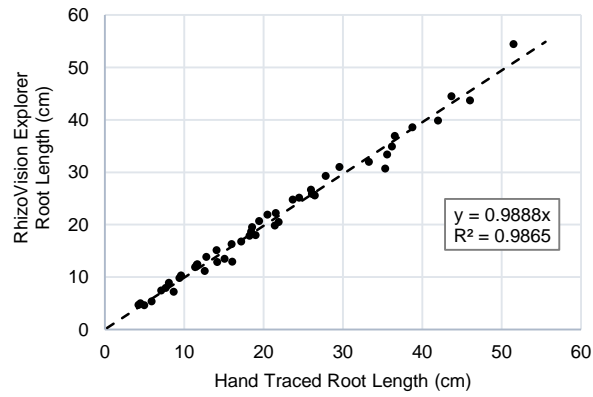

**b**

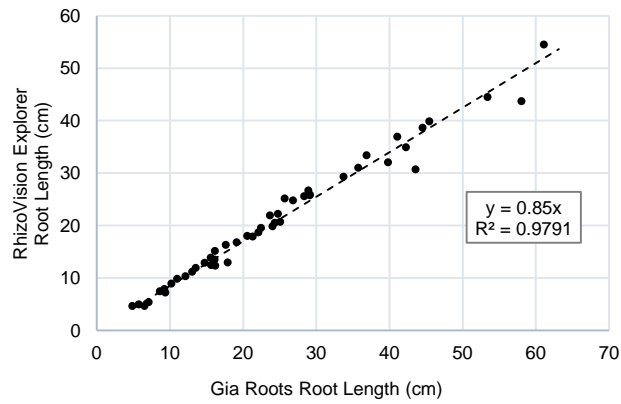

**c**

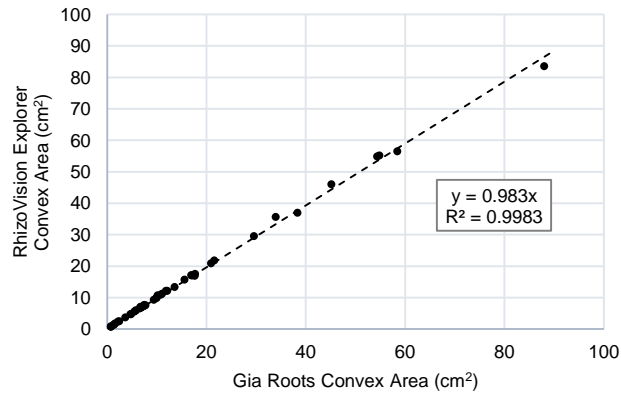

Supplement: Supplementary file 2 — Supplementary file2 Implementation of RhizoVision Explorer for phenotyping salt-stressed wheat seedlings. (a) Comparison of root lengths measured using RhizoVision Explorer with “ground truth” manual measurements on seedlings of diverse lengths and root angles. Comparison of root length (b) and convex area (c) outputs from RhizoVision Explorer with those of GiA Roots. Adaptive image thresholding was used in GiA Roots to capture as much of the root system as possible (PDF 20 KB) [file 299_2024_3206_MOESM2_ESM.pdf]

**a**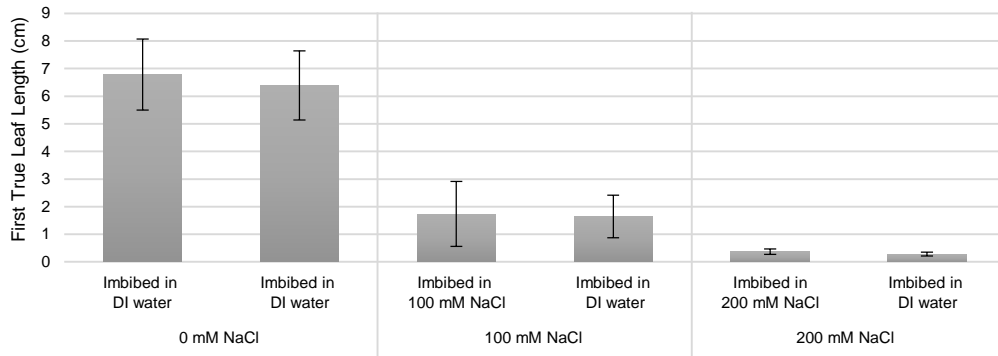**b**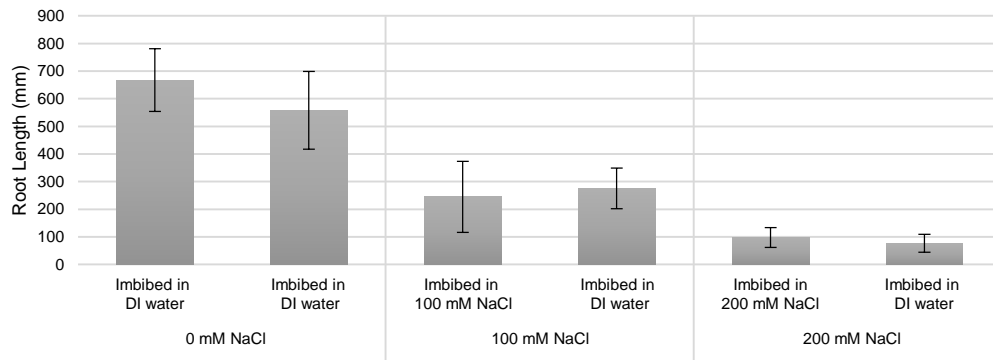**c**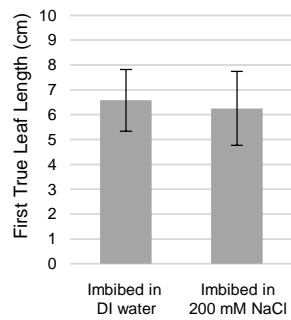**d**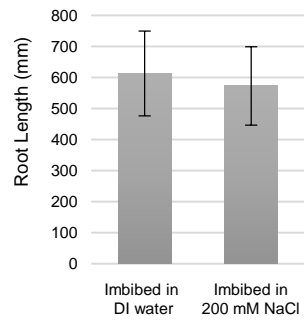**e**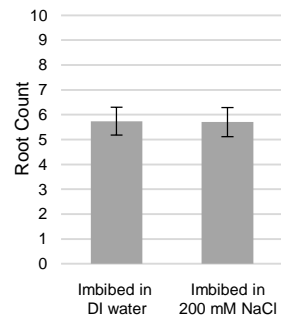

Supplement: Supplementary file 3 — Supplementary file3 Growth of seedlings when seeds were imbibed in deionized water or salt solutions. First true leaf lengths (a) and root lengths (b) when imbibed with or without salt stress [in the salt solution-soaked germination paper or in deionized (DI) water, respectively; n = 10 per treatment]. Effects of salt stress during imbibition on seeds germinating in DI water are presented for the first-true-leaf lengths (c), root lengths (d), and root count (e) (n = 20 per treatment). Error bars represent ± standard deviation. No statistically significant differences were detected within treatments (PDF 30 KB) [file 299_2024_3206_MOESM3_ESM.pdf]

a

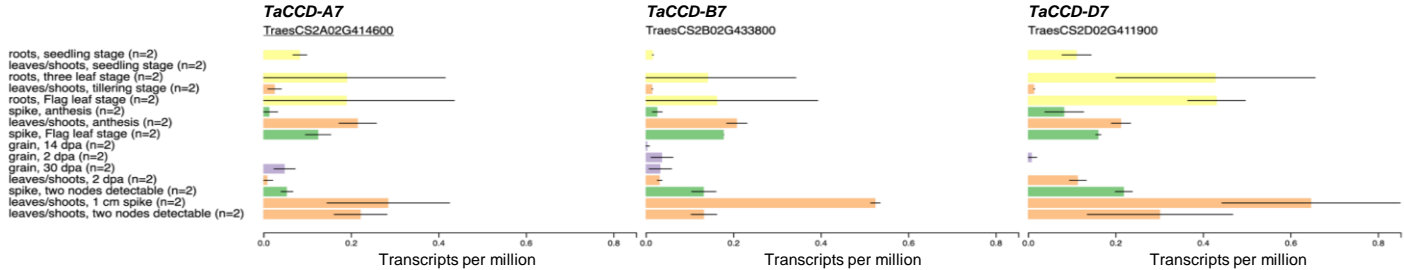

b

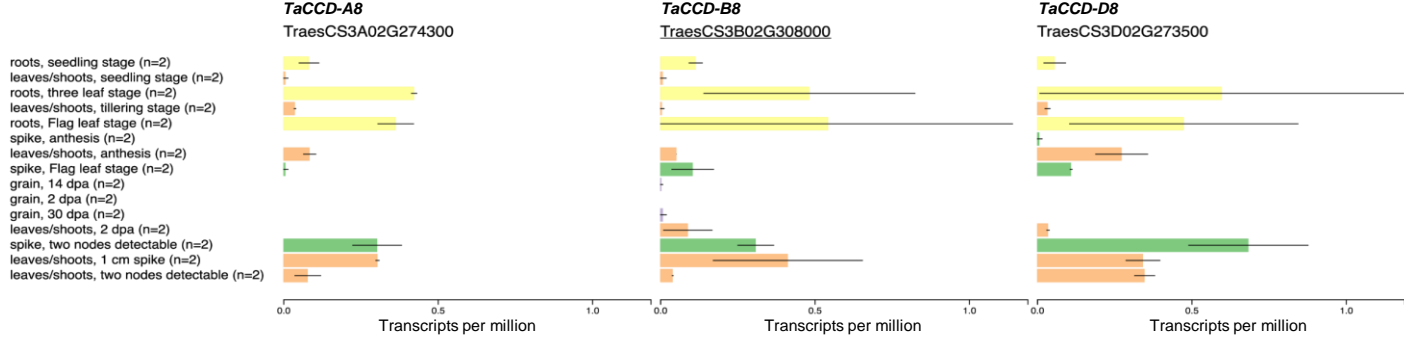

c

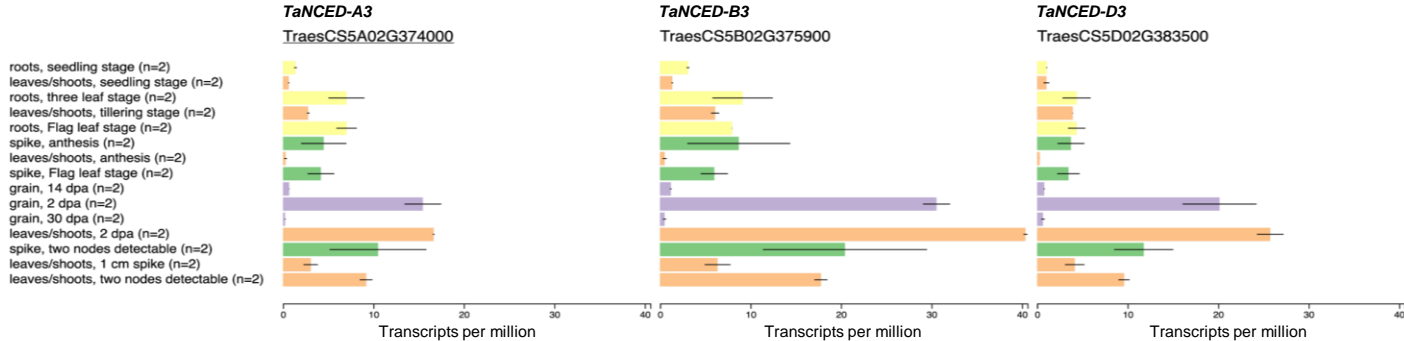

Supplement: Supplementary file 4 — Supplementary file4 In silico analysis of candidate carotenoid cleavage gene expression in hexaploid wheat across different tissue types and developmental stages. Transcript levels, in transcripts per million, are shown for the homoeologs of TaCCD7 (a), TaCCD8 (b), and TaNCED3 (c). CCD, carotenoid cleavage dioxygenase; NCED, nine-cis-epoxycarotenoid dioxygenase. The presented gene expression data were derived from the study on “Developmental time course of Chinese Spring” (Choulet et al. 2014). The variable “n” denotes the number of RNAseq libraries constructed and sequenced for each specific wheat tissue (PDF 143 KB) [file 299_2024_3206_MOESM4_ESM.pdf]

**a**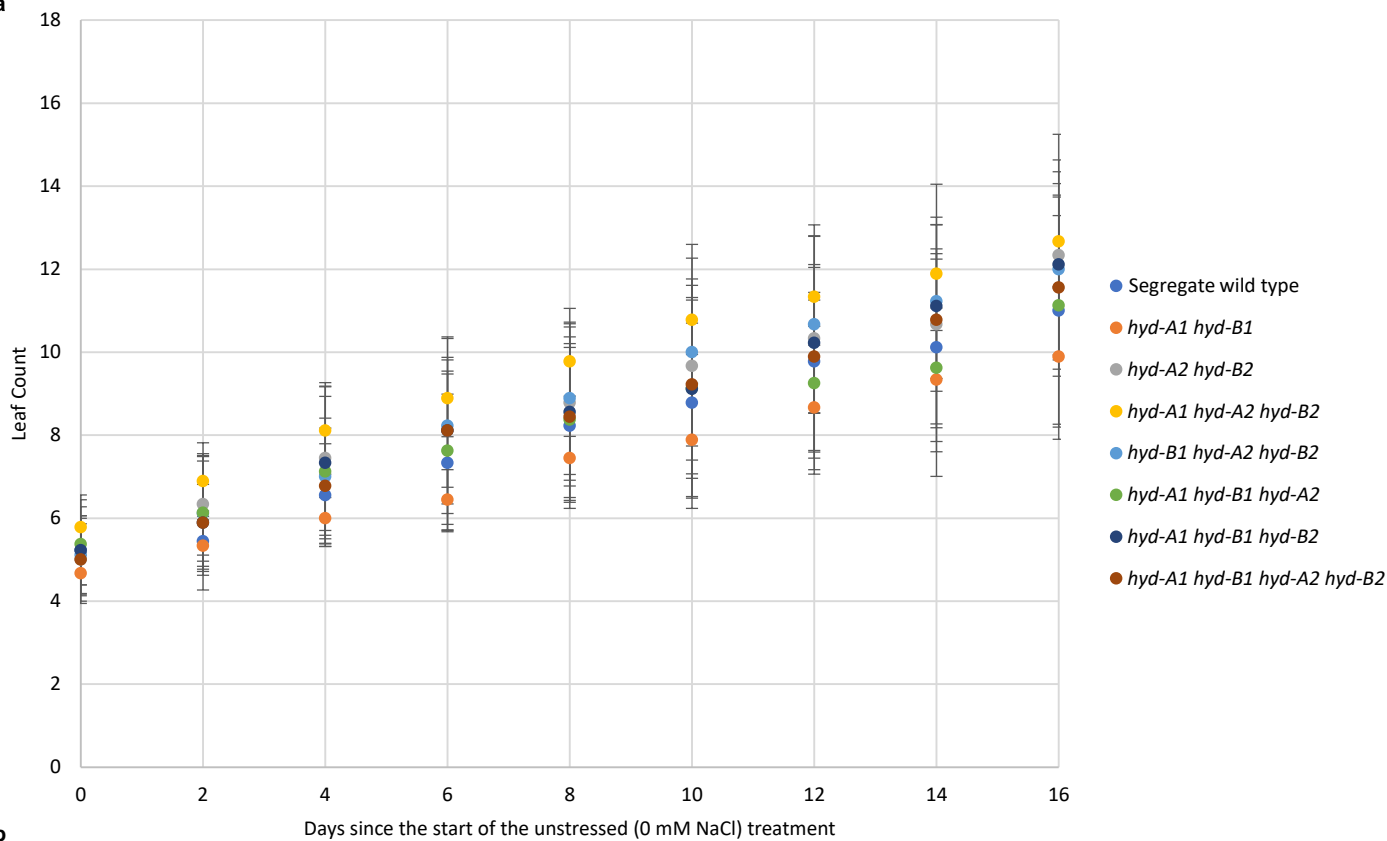**b**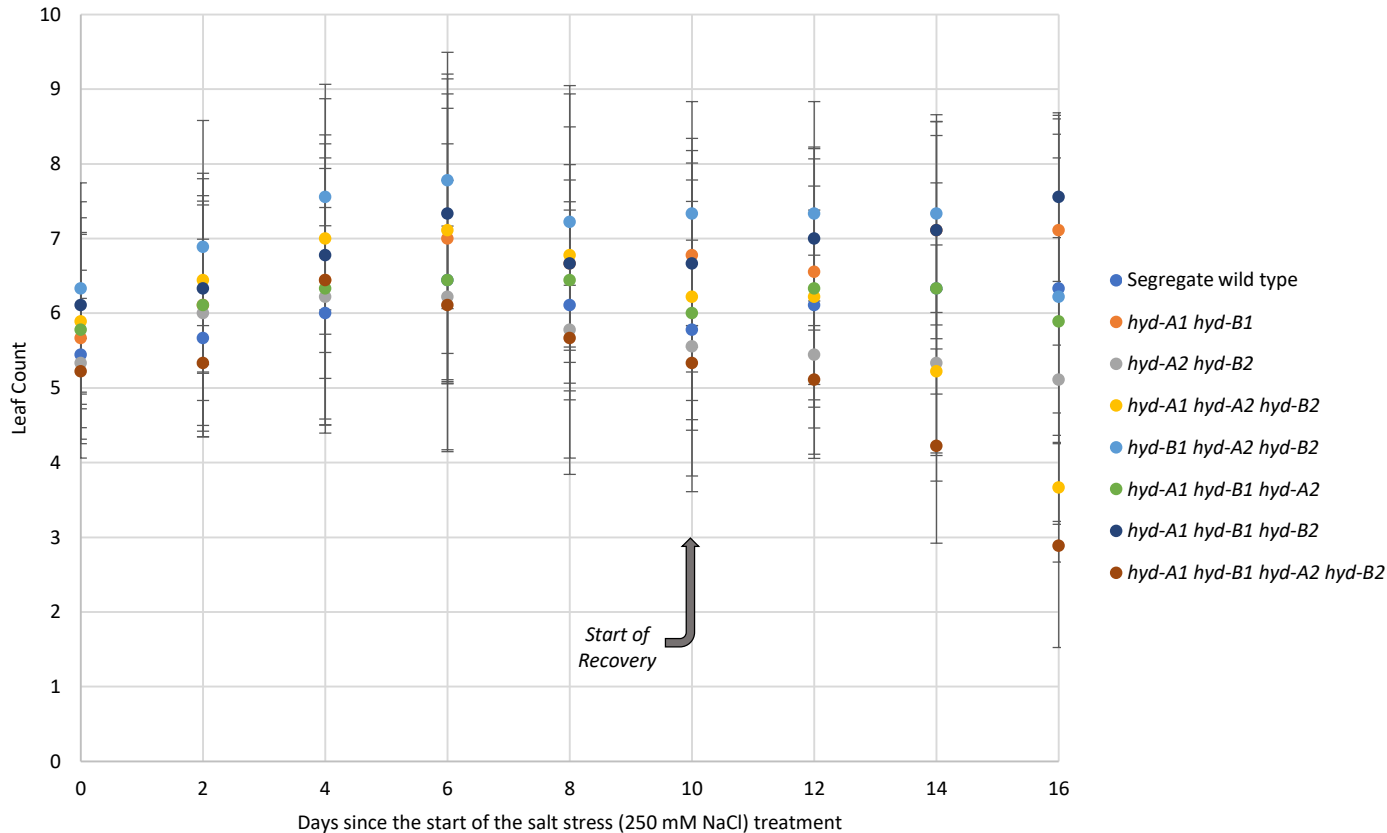

Supplement: Supplementary file 5 — Supplementary file5 Growth of tillering hyd mutant and segregate wild-type plants subjected to control (0 mM NaCl) and salt-stress treatments. Salt stress was applied at 250 mM NaCl followed by recovery in a soil-based system. Leaf counts of plants under the unstressed (0 mM NaCl) (a) and acute salt stress (250 mM NaCl) (b) treatments are shown. Error bars represent ± standard deviation (n = 9). Initiation of recovery on day 10 of the salt treatment is also denoted (PDF 348 KB) [file 299_2024_3206_MOESM5_ESM.pdf]
