# Supplementary material for: Distinct growth patterns in seedling and tillering wheat plants suggests a developmentally restricted role of HYD2 in salt-stress response
Source: Plant Cell Rep. 2024 Apr 17;43(5):119. doi: 10.1007/s00299-024-03206-x (PMC11024023; doi:10.1007/s00299-024-03206-x)
Supplement: Supplementary file 6 — Supplementary file6 (DOCX 44 KB) [file 299_2024_3206_MOESM6_ESM.docx]

## Supplementary Tables

## Table S1. Primer sequences used for real-time qPCR analysis.

| Gene | Forward (5’🡪3’) | Reverse (5’🡪3’) | Amplicon (bp) |
| --- | --- | --- | --- |
| *NCED* | GACTGCTTCTGCTTCCACCTCTG | AGGAGCCGATCACCACCAC | 73 |
| *CCD7* | GATGTGGTCGCTGCACGTC | CAGTTGTAACCGAACATCCTGTG | 111 |
| *CCD8* | ATCATCGCCGACTGCTGC | GGTAAATGCCCGGAGATTGTG | 81 |
| *Ta2291* | GCTCTCCAACAACATTGCCAAC | GCTTCTGCCTGTCACATACGC | 165 |
| *Ta54227* | CAAATACGCCATCAGGGAGAACATC | CGCTGCCGAAACCACGAGAC | 227 |

Table S2. Salt tolerance indices (STIs) for wheat seedling traits assessed across various salt concentrations. Two biological replicates with five plants each (ten plants total) were used per genotype per treatment. Mean STIs for each biological replicate were treated as individual points. Values displayed are means of two biological replicates ± standard deviation. Comparison of means was not conducted due to an insufficient number of biological replicates.

| Salt Concentration | Genotype | First True Leaf Length | Root Length | Network Area |
| --- | --- | --- | --- | --- |
| 50 mM | Segregate wild type | 0.329 ± 0.100 | 0.607 ± 0.16 | 0.588 ± 0.125 |
|  | *hyd-A1 hyd-B1* | 0.702 ± 0.156 | 0.907 ± 0.03 | 0.866 ± 0.018 |
|  | *hyd-A2 hyd-B2* | 0.582 ± 0.103 | 0.814 ± 0.255 | 0.811 ± 0.217 |
| 100 mM | Segregate wild type | 0.101 ± 0.015 | 0.326 ± 0.013 | 0.329 ± 0.013 |
|  | *hyd-A1 hyd-B1* | 0.091 ± 0.025 | 0.201 ± 0.104 | 0.183 ± 0.073 |
|  | *hyd-A2 hyd-B2* | 0.219 ± 0.149 | 0.441 ± 0.191 | 0.446 ± 0.184 |
| 150 mM | Segregate wild type | 0.070 ± 0.001 | 0.258 ± 0.013 | 0.273 ± 0.027 |
|  | *hyd-A1 hyd-B1* | 0.069 ± 0.028 | 0.161 ± 0.046 | 0.168 ± 0.048 |
|  | *hyd-A2 hyd-B2* | 0.130 ± 0.012 | 0.259 ± 0.061 | 0.283 ± 0.059 |
| 200 mM | Segregate wild type | 0.036 ± 0.005 | 0.104 ± 0.028 | 0.107 ± 0.028 |
|  | *hyd-A1 hyd-B1* | 0.056 ± 0.024 | 0.113 ± 0.023 | 0.113 ± 0.029 |
|  | *hyd-A2 hyd-B2* | 0.041 ± 0.009 | 0.101 ± 0.061 | 0.115 ± 0.078 |

Table S3. Salt tolerance indices (STIs) for seedling traits of the segregate wild-type and the *hyd* mutants. Seedling phenotypes presented in Figure 1 are bolded. Mean STIs for each biological replicate (5 plants per biological replicate) were treated as individual points. Values displayed are means of four biological replicates ± standard deviation. Statistically significant differences (*p* < 0.05) and marginally significant differences (0.05 < *p* < 0.10) between a mutant genotype and the control for a specific trait are denoted with * and †, respectively.

| Genotype | **First True Leaf Length** | **Root Count** | **Root Length** | **Root Convex Area** | Network Area | Perimeter | Volume | Surface Area |
| --- | --- | --- | --- | --- | --- | --- | --- | --- |
| Segregate wild type | 0.26 ± 0.09 | 0.86 ± 0.14 | 0.48 ± 0.07 | 0.29 ± 0.06 | 0.49 ± 0.07 | 0.46 ± 0.06 | 0.41 ± 0.06 | 0.47 ± 0.07 |
| *hyd-A1 hyd-B1* | 0.25 ± 0.08 | 0.82 ± 0.06 | 0.45 ± 0.07 | 0.34 ± 0.09 | 0.46 ± 0.08 | 0.44 ± 0.07 | 0.47 ± 0.13 | 0.47 ± 0.08 |
| *hyd-A2 hyd-B2* | 0.42 ± 0.11* | 1.07 ± 0.11* | 0.43 ± 0.14 | 0.21 ± 0.11 | 0.45 ± 0.12 | 0.41 ± 0.15 | 0.57 ± 0.15 | 0.47 ± 0.13 |
| *hyd-A1 hyd-A2 hyd-B2* | 0.39 ± 0.13* | 1.03 ± 0.07* | 0.46 ± 0.14 | 0.34 ± 0.12 | 0.49 ± 0.15 | 0.45 ± 0.14 | 0.48 ± 0.22 | 0.48 ± 0.16 |
| *hyd-B1 hyd-A2 hyd-B2* | 0.46 ± 0.06* | 1.00 ± 0.03† | 0.44 ± 0.04 | 0.24 ± 0.02 | 0.46 ± 0.04 | 0.41 ± 0.04 | 0.49 ± 0.06 | 0.47 ± 0.04 |
| *hyd-A1 hyd-B1 hyd-A2* | 0.18 ± 0.02 | 0.87 ± 0.16 | 0.37 ± 0.04 | 0.19 ± 0.05 | 0.40 ± 0.04 | 0.36 ± 0.04 | 0.40 ± 0.04 | 0.39 ± 0.03 |
| *hyd-A1 hyd-B1 hyd-B2* | 0.27 ± 0.11 | 0.92 ± 0.07 | 0.40 ± 0.09 | 0.19 ± 0.07 | 0.44 ± 0.09 | 0.37 ± 0.08 | 0.59 ± 0.15† | 0.46 ± 0.1 |
| *hyd-A1 hyd-B1 hyd-A2 hyd-B2* | 0.45 ± 0.08* | 1.00 ± 0.05† | 0.52 ± 0.08 | 0.32 ± 0.10 | 0.54 ± 0.07 | 0.51 ± 0.08 | 0.56 ± 0.14 | 0.55 ± 0.08 |
